# Effect of *Aloe vera* extract in post-burn skin repair in rats

**DOI:** 10.12688/f1000research.79538.2

**Published:** 2022-07-29

**Authors:** Lusiana Aulia, Yunita Sari Pane

**Affiliations:** 1 Faculty of Medicine, Universitas Sumatera Utara, Medan, North Sumatera, 20155, Indonesia; 2 Department of Pharmacology and Therapeutics, Universitas Sumatera Utara, Medan, North Sumatera, 20155, Indonesia

**Keywords:** burn injury, Aloe vera, macrophages, fibroblasts, epidermal thickness

## Abstract

**Background**: Burn injury is a global health problem that is most often caused by heat. A burn injury can cause high morbidity and mortality and requires high cost. Therefore, the use of plants as an herbal medicine has the potential to be developed in Indonesia. *Aloe vera *contains various active ingredients that help the wound healing process, such as glucomannan and acemannan which have the effects on the proliferation of macrophages, fibroblasts, and re-epithelialization. This study aimed to determine the effect of *Aloe vera* extract in repairing post-burn skin in rats that were analyzed from the number of macrophages, fibroblasts, and epidermal thickness.

**Methods**: This is an experimental study with a posttest-only control group design using 54 *Rattus norvegicus* Wistar strain rats. The sampling method was simple random sampling consisting of 3 groups, i.e., I. standard group, which were normal rats; II. negative control group, which were given second-degree burns and treated with gel base (without *Aloe vera* extract); III. treatment groups, which were given second-degree burns and treated with *Aloe vera* extract gel. Each group was subdivided into three smaller groups (n = 6) according to the time the lesions were evaluated. Skin tissue samplings were carried out on days 3, 14, and 21 after injury to observe the number of macrophages, fibroblasts, and epidermal thickness.

**Results**: There were significant differences in the mean number of macrophages, number of fibroblasts, and epidermal thickness in all groups ( *p*<0.05).

**Conclusion**: *Aloe vera* extract could accelerate the healing process of burns in rats.

## Introduction

Burn injury is damage to the skin or other tissues that can be caused by heat, radiation, electricity, chemicals, or friction, the most often caused by heat ( American Burn Association, 2019). Burn injury that is due to heat consists of three causes, which are burns due to hot liquids (scalds), flames (flame burns), or hot solids (contact burns) ( World Health Organization, 2018). A burn injury can be divided into four degrees based on its depth. A first-degree burn is limited to the epidermis. Second-degree burns involve both epidermis and dermis. Third-degree burns involve epidermis, dermis, and dermal appendages. Lastly, fourth-degree burns can penetrate into subdermal fat, underlying fascia, muscle, and/or bone ( ABA, 2018).

Burn injury can cause high morbidity and mortality and its treatment requires a high cost. This is because of the long-term outpatient care, such as dressing changes and some even require surgical reconstruction ( Smolle *et al.,* 2017). WHO stated that around 180,000 deaths every year are caused by burns and the majority occur in lower-middle-income countries due to a lack of adequate facilities to reduce the incidence of burns ( Menkes, 2019; WHO, 2018). According to the data from “Riset Kesehatan Dasar” RISKESDAS (Basic Health Research, Indonesia) in 2018, the proportion of burn injury in Sumatera Utara was 1% and based on the medical record data from Haji Adam Malik Hospital in Medan, North Sumatra-Indonesia, there were 353 cases of burns in 2011–2014 ( Maulana, 2014).

There are three phases of wound healing, namely the inflammatory, proliferative, and remodeling phases. The inflammatory phase is characterized by the migration of leucocytes such as macrophages to the burn site to degrade debris or microbes. Next is the proliferative phase where the process of angiogenesis, re-epithelialization, and fibroblast proliferation occurs. Lastly, the remodeling phase is characterized by collagen remodeling, wound contraction, and decreased blood flow ( Reinke & Sorg, 2012).

Several studies have shown that the use of silver sulfadiazine drug can cause side effects such as leukopenia and renal toxicity ( Chaby *et al.,* 2005; Fuller & Engler, 1988). Therefore, alternative treatments such as the use of herbal medicine are relatively safer. Several medical plants grow in Indonesia due to favorable tropical natural conditions. The information about these herbs is passed down from one generation to another, and they are commonly used in traditional medicine, forming an integral part of Indonesian cultural heritage ( Gazali *et al.,* 2013). One of the herbs that can be used for the treatment of burns is *Aloe vera.* The active chemical constituents found in *Aloe vera* such as glucomannan and acemannan can stimulate the activity and proliferation of fibroblasts. Acemannan can also increase the number of macrophages and stimulates fibroblast to produce keratinocyte growth factor-1 (KGF-1) for re-epithelialization process ( Jettanacheawchankit *et al.,* 2009; Sierra-García *et al.,* 2014; Surjushe *et al.,* 2008). This study aimed to determine the effect of *Aloe vera* extract in repairing post-burn skin in rats that were analyzed from the number of macrophages, fibroblasts, and epidermal thickness.

## Methods

This is an experimental study in an animal burn model with a posttest-only control group design. This study is reported in lines with the *in-vivo* Animal Research: Reporting guidelines ARRIVE.

### Ethics

This study was approved by the Research Ethics Committee of Universitas Sumatera Utara (No. 711/KEP/USU/2021). This study was conducted at the Pharmacology Laboratory of the Faculty of Medicine, Universitas Sumatera Utara.

### Experimental animal

Healthy male Wistar strain rats, 10–12 weeks old, weighing 150–200 g were purchased from the Department of Biology of Mathematics and Scientific Faculty, Universitas Sumatera Utara. Before starting the experiment, the animals were adapted for 7 days. Of all rats, then every three rats were put into one cage with a size 55 × 40 × 27 cm (National Centre for Replacement Refinement and Reduction of Animal in Research strongly recommended height of the cage is 26-30 cm) and covered with fine wire mesh; the base of cages was covered with rice husks as thick as 0.5-1 cm and replaced every day during the study. They were kept in 12 hours of daylight (06:00 A.M. – 06.00 P.M.) and 12 hours of dark cycle (06:00 P.M. – 06.00 A.M.) and fed with standard feed (CP 551 from PT Charoen Pokphand-Indonesia) and water was given *ad libitum.* They were kept in cages that were maintained at room temperature and humidity at normal ranges.

### Allocation and treatment groups

All animals were randomly divided into three big groups by simple random sampling. Group I (standard) consisted of normal rats, group II (negative control) was inflicted with a burn wound and treated with gel base, and group III (treatment group) was inflicted with a burn wound and treated with *Aloe vera* gel. Each group was subdivided into three smaller groups (n = 6 per group) according to the time when the lesions were evaluated. They were treated twice a day according to the groups.

The sample size was calculated according to Federer’s formula: ( Federer, 1963)

$$\left(t-1\right)\;\left(n-1\right)\geq \;15$$

t = the number of groups

n = the number of samples

n = 2.875 ≈ 3 rat/group

Considering the NC3Rs principal in experimental animal, statistical analysis necessity, and the possibility of drop out, the number of rats per group were determined to be six. All 54 rats were given a number (1–54) using a marker pen, then randomized by putting the numbers in an envelope and dividing them into 9 groups, according to the numbers taken from the envelope ( Russell and Burch, 1992).

### Wound infliction

To ensure that the rats remain in a comfortable condition, they were given anesthesia before performing the burn. The animals have anesthetized with Ketamine/Xylazine 0.2 mL by intraperitoneal injection (in accordance with the provisions of Vertebrate Animal Research). The hair at the dorsal region was shaved and cleansed with alcohol swab. Second-degree burn wound was inflicted using iron plate measuring 2×2 cm ^2^ which was warmed in boiling water for 5 minutes and placed for 30 seconds on the shaved area ( Kristyaningsih, 2016).

### *Aloe vera* extract

About 2000 g of *Aloe vera* leaves were collected. The bottom part and the serrated edges of *Aloe vera* leaves were cut and peeled to obtain the inner clear gel. About 1000 g of inner gel was weighed and washed thoroughly and cut it into small pieces. Then, the small pieces of gel and 2000 mL 70% ethanol were blended and poured into a closed container (stirred occasionally for 6 hours, then set aside for 18 hours). This mixture was then filtered to obtain the filtrate (I). The extraction process was repeated on the residue using 1000 mL of 70% ethanol to obtain another filtrate (II). The filtrate (I) and (II) were mixed and evaporated using a vacuum rotary evaporator (Heidolph Instruments GmbH & Co. KG, Germany) at 40 ^0^C to obtain a thick extract.

### Gel preparation

• Gel base
  About 400 cc of distilled water was taken into the mortar, then 12 g of 3% Sodium-Carboxymethyl cellulose (Na-CMC) powder was evenly added. It was closed and set it aside for 15 minutes. After which it was ground and 4 g of 1% glycerin was added until a homogeneous gel base was formed.
• *Aloe vera* gel
  Put 40 g of *Aloe vera* extract into the mortar then add 360 g of gel base gradually while grinding it until homogeneous. So, the concentration of the *aloe vera* contained 10% of the total weight of the gel.

### Hematoxylin and eosin (H&E) staining

Six animals of each groups were sacrificed on the 3 ^rd^, 14 ^th^, and 21 ^st^ days after injury by injecting 100 mg/kg ketamine intraperitoneally. Then, the injured tissues were carefully dissected and fixed with 10% formalin. All samples were taken to Anatomic Pathology Laboratory of the Faculty of Medicine, Universitas Sumatera Utara for H&E staining. The number of macrophages and fibroblasts, and epidermal thickness was evaluated using Olympus CX21 light microscope with 400× magnification and three fields of view.

### Statistical analysis

Data were analyzed using SPSS version 20 (RRID:SCR_019096) with one-way ANOVA test. If there were significant differences (p<0.05), then LSD test was conducted to determine the differences among groups. Data were presented as mean ± SD.

## Results

**Figure 1.**
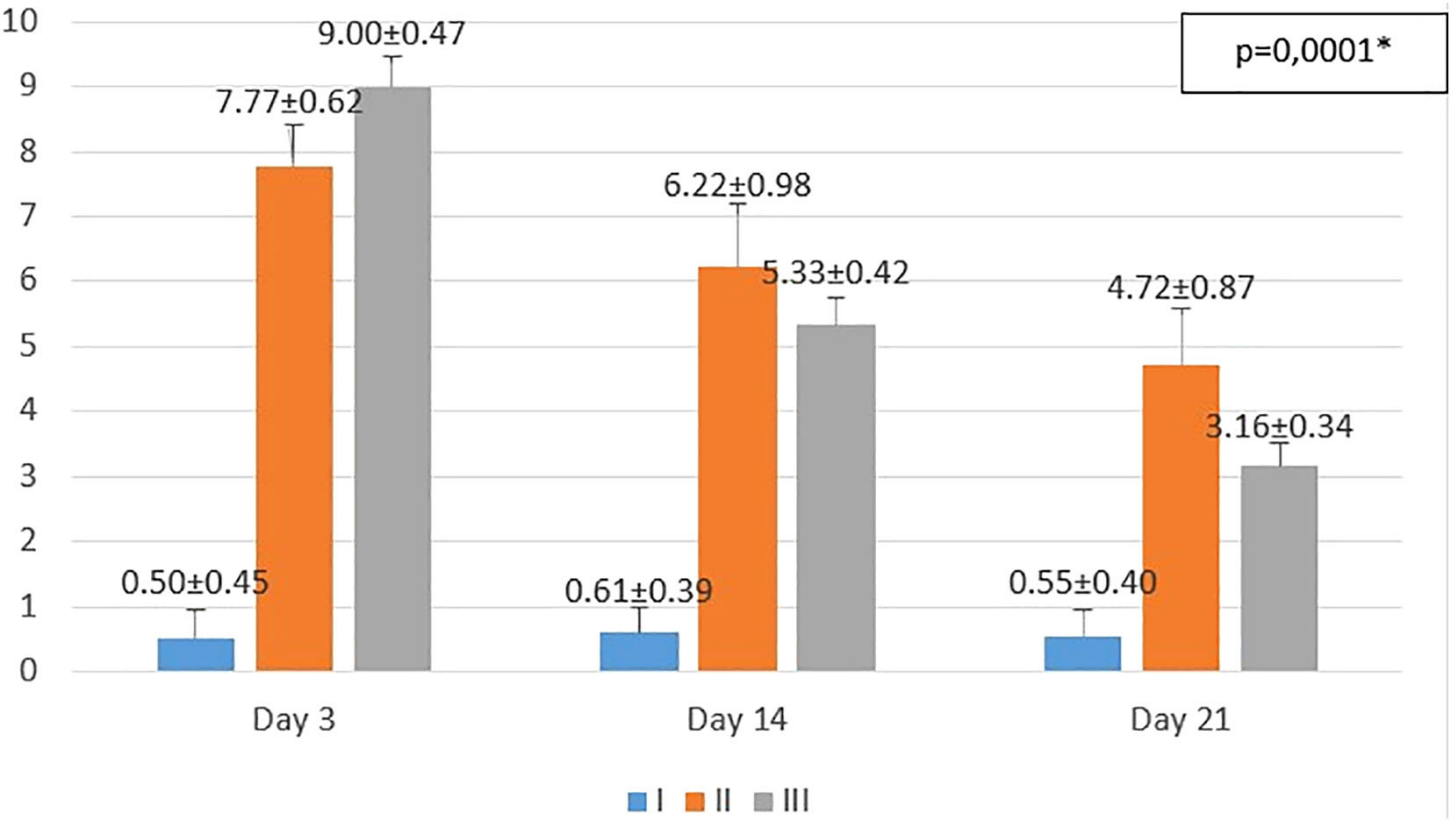
Mean number of macrophages. I (standard), II (negative control), III (treatment). *p<0.05 statistically analysis by ANOVA showed significant difference.

**Figure 2.**
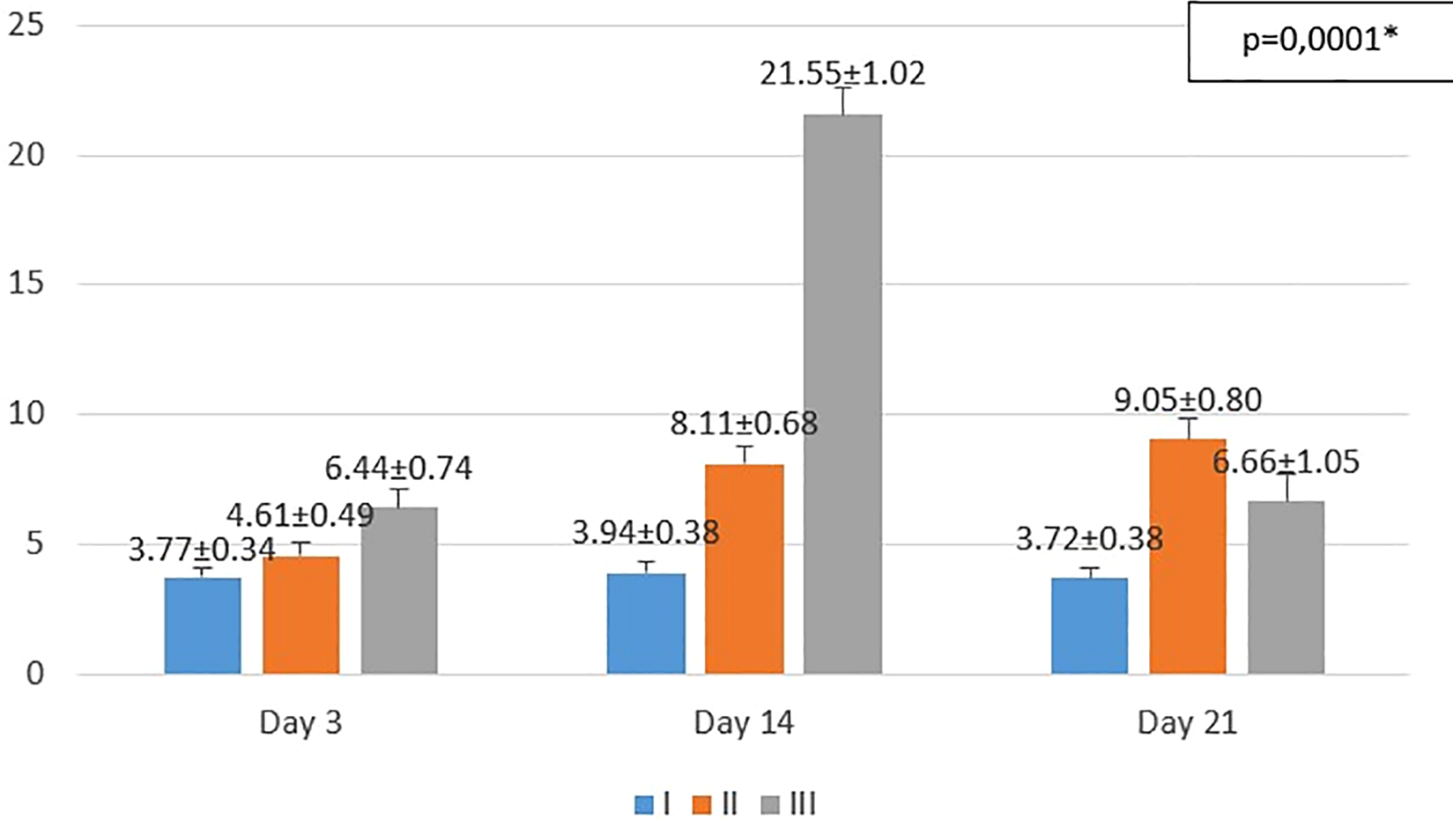
Mean number of fibroblasts I (standard), II (negative control), III (treatment) *p<0.05 statistically analysis by ANOVA showed significant difference.

**Figure 3.**
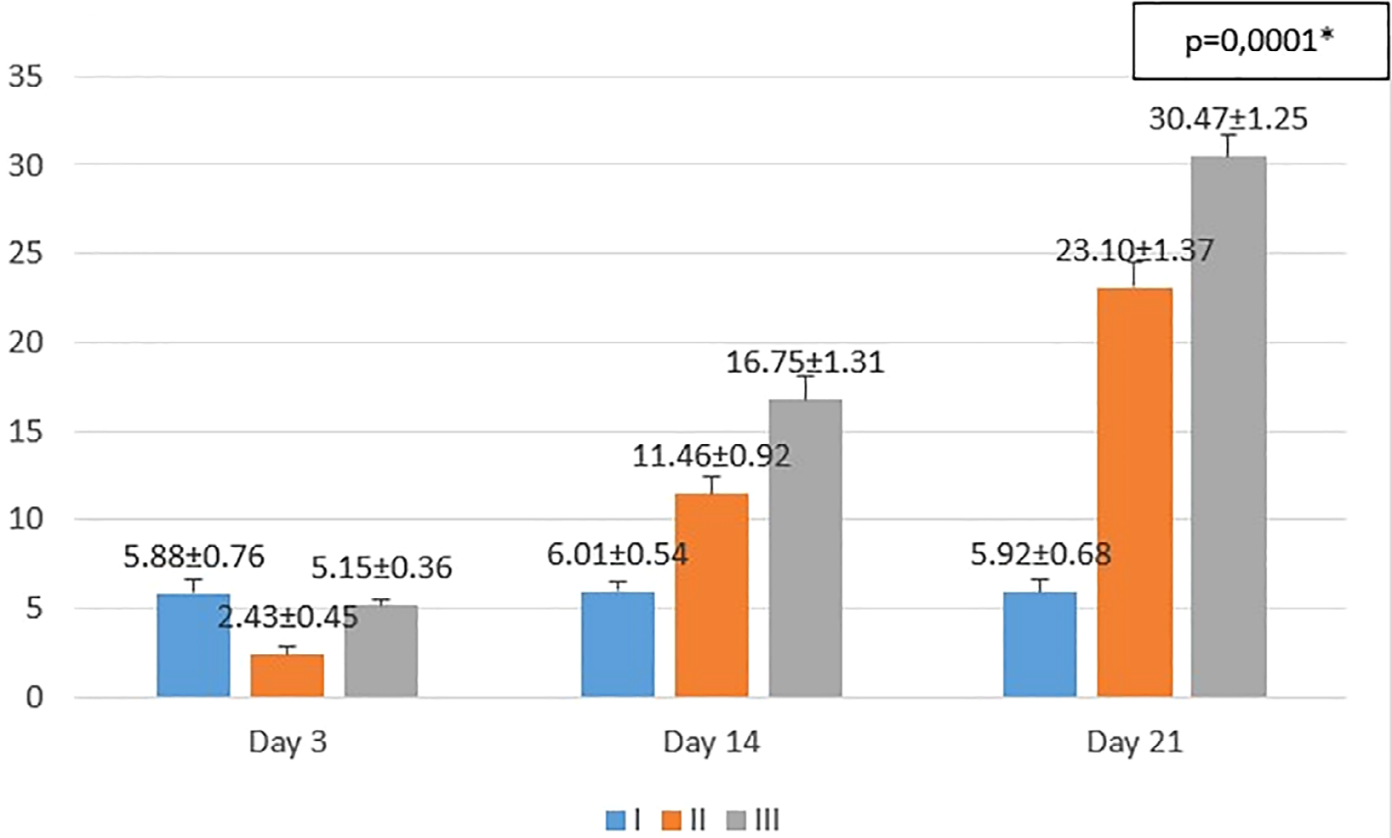
Mean number of epidermal thickness. I (standard), II (negative control), III (treatment). *p<0.05 statistically analysis by ANOVA showed significant difference.

**Figure 4.**
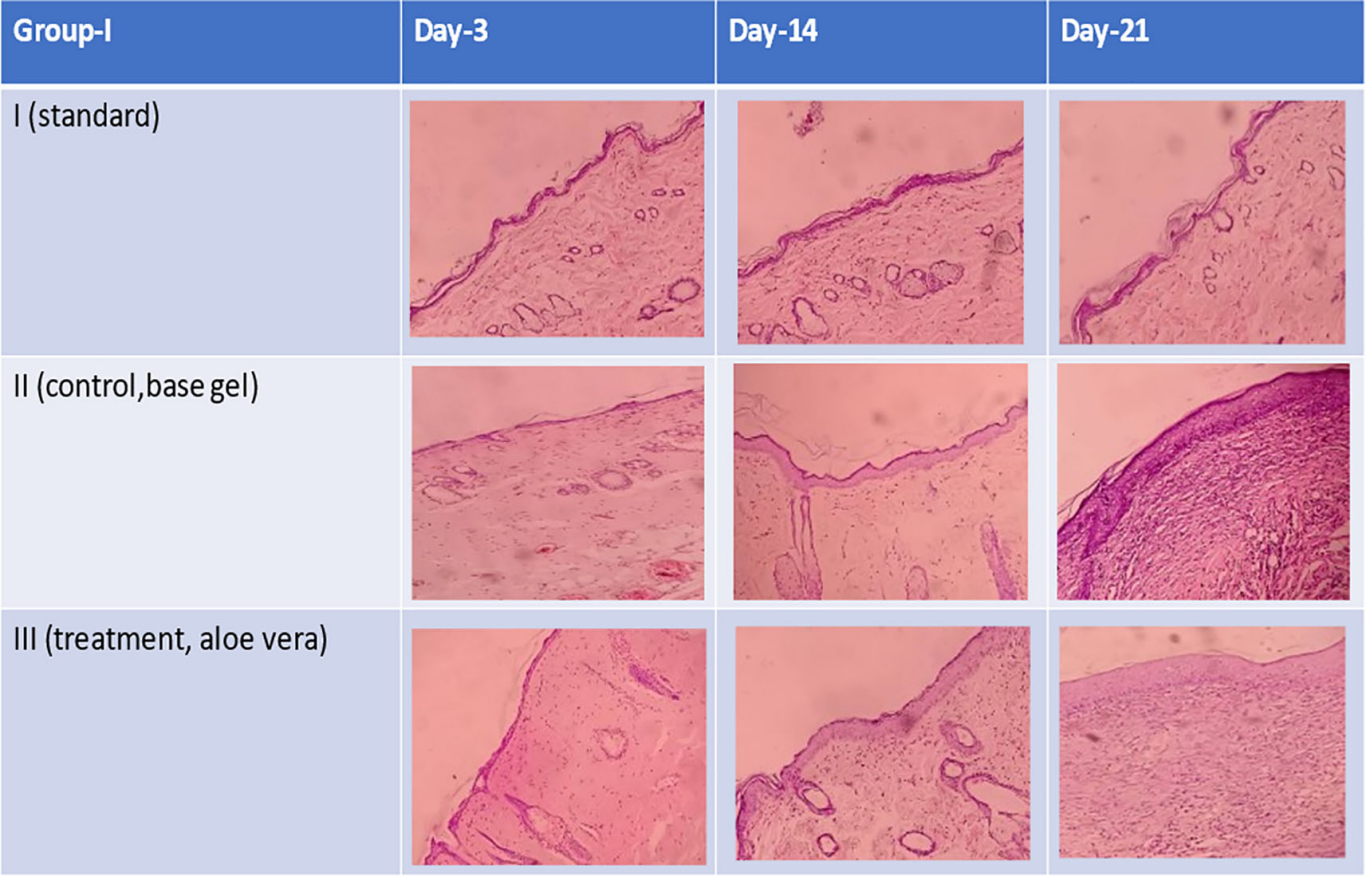
There was a change in epidermal thickness on days 3, 14 and 21. The thickest epidermal layer was found in group III (treatment, *aloe vera*) compared to group II (negative control). This could be due to the fact that *aloe vera* gel was better in the wound repair process than the other groups. The thickness of the epidermis in groups II and III was also thicker than in the control group. This can happen because there is still a wound repair process in groups II and III.

**Figure 5.**
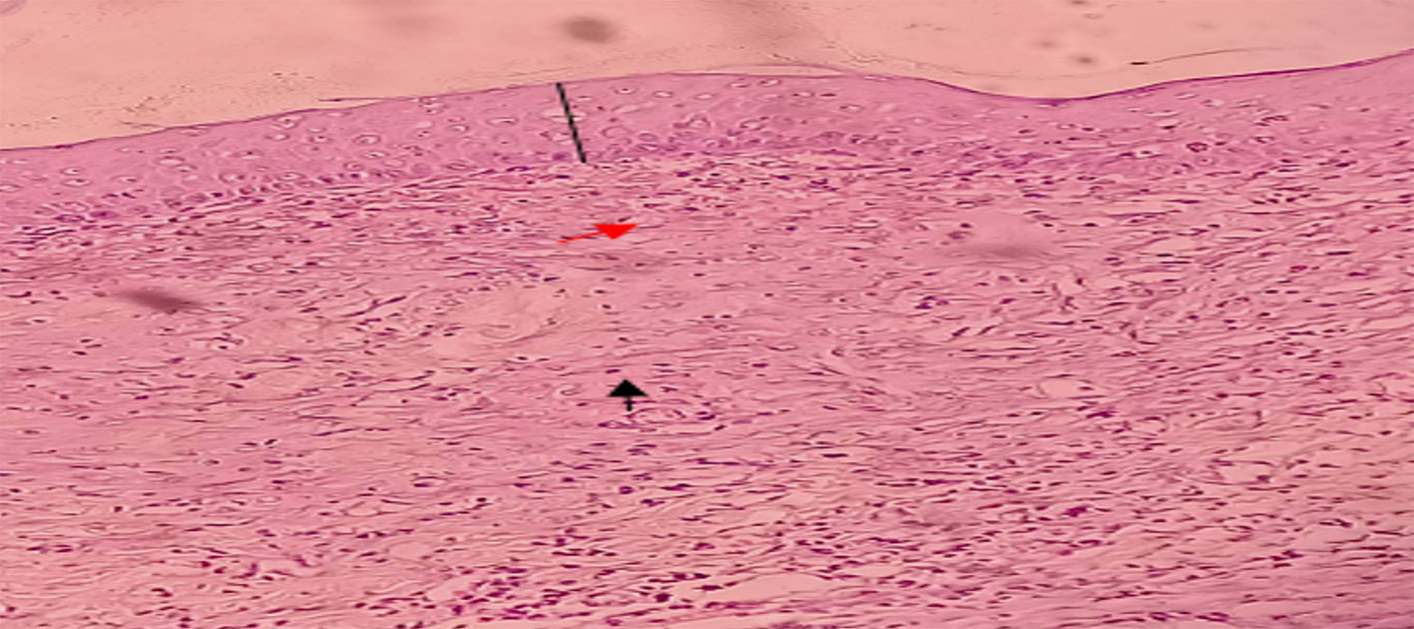
The histopathological description of macrophages ( 
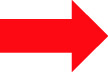
) fibroblast ( 
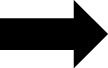
) and epidermal thickness ( 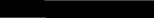).

The results obtained from the study are shown in Figures 1− 3. The comparison of the changes in the thickness of the epidermis that occurs is shown in Figure 4. The explanation of the histopathological images obtained for macrophages, fibroblasts, and epidermal thickness is shown in Figure 5. From the one-way ANOVA results, there were significant differences in the mean number of macrophages and fibroblasts, and epidermal thickness in all groups (p<0.05). Then, the least Significant Difference (LSD) test was conducted to determine the differences among groups.

On the 3 ^rd^ day after injury, the highest mean number of macrophages were seen in the treatment group (III), followed by the negative control group (II), and followed by the standard group (I). From the *post hoc* test results, it appeared that there were significant differences in the mean number of macrophages (p<0.05) in groups I-3 vs II-3 (0.50 ± 0.45 vs 7.77 ± 0.62; p=0.0001), group I-3 vs III-3 (0.50 ± 0.45 vs 9.00 ± 0.47; p=0.0001), and group II-3 vs III-3 (7.77 ± 0.62 vs 9.00 ± 0.47; p=0.001) (see Figure 1).

On the 14 ^th^ day after injury, the highest mean number of macrophages were seen in the negative control group (II), followed by the treatment group (III), and followed by the standard group (I). From the *post hoc* test results, it appeared that there were significant differences in the mean number of macrophages (p<0.05) in groups I-14 vs II-14 (0.61 ± 0.39 vs 6.22 ± 0.98; p=0.0001), group I-14 vs. III-14 (0.61 ± 0.39 vs. 5.33 ± 0.42; p=0.0001), and group II-14 vs. III-14 (6.22 ± 0.98 vs 5.33 ± 0.42; p=0.013) (see Figure 1).

On day 21 ^st^ day after injury, the highest mean number of macrophages were seen in the negative control group (II), followed by the treatment group (III), and followed by the standard group (I). From the *post hoc* test results, it appeared that there were significant differences in the mean number of macrophages (p<0.05) in groups I-21 vs II-21 (0.55 ± 0.40 vs 4.72 ± 0.87; p=0.0001), group I-21 vs III-21 (0.55 ± 0.40 vs 3.16 ± 0.34; p=0.0001), and group II-14 vs III-14 (6.22 ± 0.98 vs 3.16 ± 0.34; p=0.0001) (see Figure 1).

On the 3 ^rd^ day after injury, the highest mean number of fibroblasts were seen in the treatment group (III), followed by the negative control group (II), and followed by the standard group (I). From the *post hoc* test results, it appeared that there were significant differences in the mean number of fibroblasts (p<0.05) in groups I-3 vs II-3 (3.77 ± 0.34 vs. 4.61 ± 0.49; p=0.047), group I-3 vs III-3 (3.77 ± 0.34 vs 6.44 ± 0.74; p=0.0001), and group II-3 vs III-3 (4.61 ± 0.49 vs 6.44 ± 0.74; p=0.0001) (see Figure 2).

On the 14 ^th^ day after injury, the highest mean number of fibroblasts were seen in the treatment group (III), followed by the negative control group (II), and followed by the standard group (I). From the *post hoc* test results, it appeared that there were significant differences in the mean number of fibroblasts (p<0.05) in groups I-14 vs II-14 (3.94 ± 0.38 vs 8.11 ± 0.68; p=0.0001), group I-14 vs III-14 (3.94 ± 0.38 vs 21.55 ± 1.02; p=0.0001), and group II-14 vs III-14 (8.11 ± 0.68 vs 21.55 ± 1.02; p=0.0001) (see Figure 2).

On the 21 ^st^ day after injury, the highest mean number of fibroblasts were seen in the negative control group (II), followed by the treatment group (III), and followed by the standard group (I). From the *post hoc* test results, it appeared that there were significant differences in the mean number of fibroblasts (p<0.05) in groups I-21 vs II-21 (3.72 ± 0.38 vs 9.05 ± 0.80; p=0.0001), group I-21 vs III-21 (3.72 ± 0.38 vs 6.66 ± 1.05; p=0.0001), and group II-21 vs III-21 (9.05 ± 0.80 vs 6.66 ± 1.05; p=0.0001) (see Figure 2).

On the 3 ^rd^ day after injury, the mean epidermal thickness was highest in the standard group (I), followed by the treatment group (III), and followed by the negative control group (II). From the *post hoc* test results, it appeared that there were significant differences in the mean epidermal thickness (p<0.05) in groups I-3 vs II-3 (5.88 ± 0.76 vs 2.43 ± 0.45; p=0.0001), group II-3 vs III-3 (2.43 ± 0.45 vs 5.15 ± 0.36; p=0.0001), while group I-3 vs. III-3 (5.88 ± 0.76 vs 5.15 ± 0.36; p=0.182) did not show a significant result (see Figure 3).

On the 14 ^th^ day after injury, the mean epidermal thickness was highest in the treatment group (III), followed by the negative control group (II), and followed by the standard group (I). From the *post hoc* test results, it appeared that there were significant differences in the mean epidermal thickness (p<0.05) in groups I-14 vs II-14 (6.01 ± 0.54 vs 11.46 ± 0.92; p=0.0001), group I-14 vs III-14 (6.01 ± 0.54 vs 16.75 ± 1.31; p=0.0001), and group II-14 vs III-14 (11.46 ± 0.92 vs 16.75 ± 1.31; p=0.0001) (see Figure 3).

On the 21 ^st^ day after injury, the mean epidermal thickness was highest in the treatment group (III), followed by the negative control group (II), and followed by the standard group (I). From the *post hoc* test results, it appeared that there were significant differences in the mean epidermal thickness (p<0.05) in groups I-21 vs II-21 (5.92 ± 0.68 vs 23.10 ± 1.37; p=0.0001), group I-21 vs III-21 (5.92 ± 0.68 vs 30.47 ± 1.25; p=0.0001), and group II-21 vs III-21 (23.10 ± 1.37 vs 30.47 ± 1.25; p=0.0001) (see Figure 3).

## Discussion

Macrophages are cells that multiply when there is a wound to phagocytose debris and bacteria and they are the predominant cells on the 3 ^rd^ day post wound infliction ( Gurtner & Wong, 2013). This study showed that both negative control and treatment groups had more number of macrophages than standard group on the 3 ^rd^ day after injury. On the same day, the treatment group had more macrophages than negative control group because there is acemannan in aloe gel that could increase the number of macrophages ( Sierra-García *et al.,* 2014). The results of this study showed that there was reduction in number of macrophages in the treatment group than the negative control group on the 14 ^th^ and 21 ^st^ days after injury. Souza *et al. (*2017) reported a decrease in the number of macrophages in burn rats treated with 1% silver sulfadiazine.

In this study, both the 3 ^rd^ and 14 ^th^ days after injury demonstrated a higher number of fibroblasts in the treatment group than the negative control group. This might have happened because of interaction between glucomannan in aloe gel and gibberellin, a growth hormone, with growth factor receptors on fibroblast. This interaction stimulated fibroblast activity and proliferation that could increase collagen synthesis ( Chithra *et al.,* 1998; Surjushe *et al.,* 2008). Besides that, there was also acemannan in aloe gel which could increase the proliferation of fibroblasts ( Xing *et al.,* 2014). Fibroblasts are cells that will increase their migration during the proliferation phase of wound healing. Therefore, this study showed highest number of fibroblasts on the 14 ^th^ day after injury in the treatment group which was earlier than the negative control group that had the highest number on the 21 ^st^ day after injury. At some point during the proliferation phase, there may be apoptosis of fibroblast when collagen matrix has filled the wound cavity ( Gurtner & Wong, 2013). Therefore, there was a reduction in the number of fibroblasts on the 21 ^st^ day after injury in the treatment group.

Re-epithelialization occurs right after injury that consists of migration, proliferation, and differentiation of keratinocytes ( Gurtner & Wong, 2013; Isrofah and Afandi, 2015). Acemannan could stimulate fibroblast to release keratinocyte growth factor-1 (KGF-1) that could hasten the re-epithelialization process by keratinocytes which could increase the epidermal thickness ( Jettanacheawchankit *et al.,* 2009). The result of this study showed that there was an increase in the epidermal thickness in both, negative control and treatment groups from the 3 ^rd^ day to the 21 ^st^ day after injury. But the treatment group had thicker epidermis than the negative control group which might be caused by acemannan. An *in vitro* study conducted by Teplicki *et al. (*2018) demonstrated that *Aloe vera* could speed up the migration and proliferation of keratinocytes. Another study conducted by Atiba *et al. (*2015) showed that *Aloe vera* could enhance the re-epithelialization process in corneal alkali burn in normal and diabetic rats.

Histopathological appearance of HE staining in burn model mice, group-I (standard/normal) without treatment from days 3, 14, and 21 did not change the number of macrophages and fibroblasts, and also there were no shown significant differences in epidermal thickening (see Figures 1, 2, 3 and 4). In group-II (negative control) tracing the migration of macrophages and fibroblasts on days 3, 14, and 21 were not significantly different, but the thickness of the epidermis could be seen from the data and the histopathological appearance seemed to increase ( Figures 1, 2 and 4). This is because the physiological healing process is still going on without intervention, only given a topical gel base (see Figures 3 and 4). Meanwhile in group 3 (see Figures 3 and 4) the healing process occurred faster with greater epidermal thickening compared to untreated burn rats (negative control). This may be due to the fact that the remodeling phase runs faster on Group III than Group II without *aloe vera* treatment (negative control). This is suggested due to the presence of acemannan substances that play a role in increasing epidermal thickening, this is in line with research by Reinke and Sorg (2012).

## Conclusion

This study showed that *Aloe vera* extract could accelerate the healing process of burns in rats.

## Data availability

### Underlying data

Figshare: Effect of *Aloe Vera* in post-burn skin repair in rats

https://doi.org/10.6084/m9.figshare.17194973.v1

### Reporting guidelines

Figshare: ARRIVE checklist for ‘Effect of Aloe Vera in Post-Burn Skin Repair in Rats’ ( Aulia and Pane, 2022)

https://doi.org/10.6084/m9.figshare.17194973.v1

Data are available under the terms of the Creative Commons Zero “No rights reserved” data waiver (CC0 1.0 Public domain dedication).

## Author contributions

Aulia L: Conceptualization, Formal Analysis, Investigation, Writing – Original Draft Preparation; Pane YS: Conceptualization, Formal Analysis, Writing – Original Draft Preparation, Writing – Review & Editing
