# Supplementary material for: Effect of Aloe vera extract in post-burn skin repair in rats
Source: F1000Res. 2023 Feb 28;11:168. Originally published 2022 Feb 11. [Version 3] doi: 10.12688/f1000research.79538.3 (PMC10160790; doi:10.12688/f1000research.79538.3)
Supplement: Peer review history

# Peer review: Reviewer response for version 3

**DOI:** 10.5256/f1000research.144246.r145983

**Published:** 2023-05-04

**Authors:** Fatima Ali

**Affiliations:** 1 Applications of Stem Cells in Cancer Therapy: A Literature Based Studies, University of Lahore, Lahore, Pakistan

Greeting of a day!

 i go through the revised version and stand it approved.

Is the work clearly and accurately presented and does it cite the current literature?

Partly

If applicable, is the statistical analysis and its interpretation appropriate?

Partly

Are all the source data underlying the results available to ensure full reproducibility?

Yes

Is the study design appropriate and is the work technically sound?

Partly

Are the conclusions drawn adequately supported by the results?

Partly

Are sufficient details of methods and analysis provided to allow replication by others?

No

Reviewer Expertise:

Molecular Biology and stem cell

I confirm that I have read this submission and believe that I have an appropriate level of expertise to confirm that it is of an acceptable scientific standard.

# Peer review: Reviewer response for version 2

**DOI:** 10.5256/f1000research.135733.r157960

**Published:** 2023-02-01

**Authors:** Reza Vaghardoost

**Affiliations:** 1 Department of Plastic and Reconstructive Surgery, Burn Research Center, Iran University of Medical Sciences, Tehran, Iran

Major concerns:

1. In the method section, the authors mentioned that they divided the rats into 3 groups, group 1 no treatment, group 2 with gel base and group 3 with *Aloe Vera* gel. Why did they name group 1 as standard group? What do they mean by "standard"?
2. This study lacks the visual observation with camera and authors did not show any images of the appearance of the wounds and wound improvement. This is a major concern and needs to be addressed.
3. The conclusion is a bold statement. What are the next steps?
4. What are the limitations of the study?

Is the work clearly and accurately presented and does it cite the current literature?

Yes

If applicable, is the statistical analysis and its interpretation appropriate?

Partly

Are all the source data underlying the results available to ensure full reproducibility?

Partly

Is the study design appropriate and is the work technically sound?

Yes

Are the conclusions drawn adequately supported by the results?

No

Are sufficient details of methods and analysis provided to allow replication by others?

Partly

Reviewer Expertise:

Burn injuries and reconstructive surgery

I confirm that I have read this submission and believe that I have an appropriate level of expertise to state that I do not consider it to be of an acceptable scientific standard, for reasons outlined above.

# Response

**Published:** 2023-02-09

**Authors:** Yunita Sari Pane

**Affiliations:** Universitas Sumatera Utara, Indonesia

**Question No 1**. In the method section, the authors mentioned that they divided the rats into 3 groups, group 1 without treatment, group 2 with gel base and group 3 with Aloe Vera gel. Why did they name group 1 as standard group? What do they mean by "standard"?

**Answer:**

 Naming group 1 as the standard group because group I is a group consisting of normal rat samples without being given burns or treatment only as a comparison to normal skin histology.

 This research is a true experimental study which has a control variable and the sample is randomly selected.

 At the beginning of the study we divided 54 rats into 3 large groups (group-I, II and III) with 3 small groups in them (day 3, 14 and 21) of each group @ 6 rats, namely:

- Group I consisted of 18 rats divided into 3 small groups of 6 rats each. This group will be executed and their skin tissue will be taken on day-3, day-14 and day-21. The skin tissue taken was then examined histopathologically and used as a control skin of NORMAL rats without being treated for BURN. Therefore  we call Group I  as the "STANDARD GROUP".
- Group II consisted of 18 rats divided into 3 small groups of 6 rats each which were treated with BURN WOUNDS on the back of each rat, and every day they were smeared with gel base (gel without active substance) to see how the burn wound healed physiologically . We named  this Group II the "NEGATIVE CONTROL GROUP" because it was not given any treatment.
- Whereas group III consisted of 18 rats divided into 3 small groups of 6 rats each which were treated with BURNS on the back of each rat, and smeared with Aloe vera gel (a gel with the active ingredient Aloe vera herb) every day to see how the process is progressed to healing burns with Aloe vera treatment. We call this Group III the "TREATMENT GROUP" because they were given the Aloe vera gel treatment.

**Question No 2. **This study lacks visual observation with a camera and the authors do not show a picture of the appearance of the wound and wound repair. This is a major concern and needs to be addressed.

**Answer:**

 See Figure 6 in the new version.

**Question No 3. **The conclusion is a bold statement. What's the next step?

**Answer:**

 See the conclusion in the new version.

**Question No 4. **What are the limitations of the study?

**Answer:**

- Application of gel to burns in rats that cannot be controlled because rats can lick/clean it even though it has been prepared by making burns on the rat's back which is difficult for them to reach compared to the stomach, legs and other areas.
- The rat rubbed the wound so that the application of Aloe vera gel became less/ineffective.
- The limited time of this research makes this research must be completed within the specified time.

# Peer review: Reviewer response for version 2

**DOI:** 10.5256/f1000research.135733.r145982

**Published:** 2022-08-01

**Authors:** Gusbakti Rusip

**Affiliations:** 1 Department of Physiology, Faculty of Medicine, Universitas Prima Indonesia, Sumatera Utara, Indonesia

The answer from the author is by the response; the manuscript is perfect. I approve the article

Is the work clearly and accurately presented and does it cite the current literature?

Yes

If applicable, is the statistical analysis and its interpretation appropriate?

Partly

Are all the source data underlying the results available to ensure full reproducibility?

Yes

Is the study design appropriate and is the work technically sound?

Yes

Are the conclusions drawn adequately supported by the results?

Yes

Are sufficient details of methods and analysis provided to allow replication by others?

Yes

Reviewer Expertise:

Physiology human, exercise, and much research-related use plants as herbal medicine

I confirm that I have read this submission and believe that I have an appropriate level of expertise to confirm that it is of an acceptable scientific standard.

# Peer review: Reviewer response for version 1

**DOI:** 10.5256/f1000research.83532.r128998

**Published:** 2022-04-28

**Authors:** Fatima Ali

**Affiliations:** 1 Applications of Stem Cells in Cancer Therapy: A Literature Based Studies, University of Lahore, Lahore, Pakistan

Major:

1. The authors mainly focuses on wound repair after burn injury but fail to show the result. Only the data of fibroblast and macrophages did not justify the whole questions? To answer this question author must show picture of wound healing, histopathological picture, wound healing area,
2.  How authors say that the size of  2×2 cm2 is reduced? Author must show the data about the wound healing in term of wound healing picture and wound healing area?
3. Images of a H&E staining should be provided and correlate it with number of fibroblast and other mentioned cells.
4. Improve the scientific language.

Is the work clearly and accurately presented and does it cite the current literature?

Partly

If applicable, is the statistical analysis and its interpretation appropriate?

Partly

Are all the source data underlying the results available to ensure full reproducibility?

Yes

Is the study design appropriate and is the work technically sound?

Partly

Are the conclusions drawn adequately supported by the results?

Partly

Are sufficient details of methods and analysis provided to allow replication by others?

No

Reviewer Expertise:

Stem cell

I confirm that I have read this submission and believe that I have an appropriate level of expertise to state that I do not consider it to be of an acceptable scientific standard, for reasons outlined above.

# Response

**Published:** 2022-06-30

**Authors:** Yunita Sari Pane

**Affiliations:** Universitas Sumatera Utara, Indonesia

Dear Fatima Ali,

 Thank you for the comments on our manuscript. I would like to highlight some particulars regarding the work we have done according to the comments:

1. We have revised the results section regarding histopathological images as microscopic parameters. So, we don't need to show a picture of wound healing (macroscopic parameters), because it isn’t in accordance with the aim of this study i.e: mean number of macrophages, number of fibroblasts, and epidermal thickness. 
2. In this study, there is no statement of a reduction in burns. As for the information about 2x2cm2, it is the area of ​​​​the wound that was determined in this study ( see in wound inflection part of the method).
3. In the revision, we have added histopathological images of macrophages, fibroblasts, and epidermal thickening. the explanation histopathological picture can be correlated with pictures 1, 2, and 3 (see results section).
4. Thank you for your suggestion, we will try to improve our ability in scientific language.

# Peer review: Reviewer response for version 1

**DOI:** 10.5256/f1000research.83532.r123296

**Published:** 2022-02-28

**Authors:** Gusbakti Rusip

**Affiliations:** 1 Department of Physiology, Faculty of Medicine, Universitas Prima Indonesia, Sumatera Utara, Indonesia

The manuscript by Lusiana Aulia and Yunita Sari Pane presents the intervening Effect of *Aloe vera* extract in post-burn skin repair in rats. In general, I find this manuscript to make an important contribution in developing palatable and superior bioactive compounds in *Aloe vera *containing various active ingredients that help the wound healing process, such as glucomannan and acemannan. However, I have outlined below some of the issues that need to be addressed prior to indexing, and some minor comments and suggestions to further improve the quality of the manuscript.

**Major**

1. In the abstract and introduction, the author states that one of the objectives of this study is to improve post-burn skin with the intervention of *Aloe vera* extract. However, histopathological description of the healing process of burns is not presented and discussed.
2. The method part is not clear. It is not clear the analytical method for macrophage and fibroblast counts and the analytical formula is not specified, the authors need to explain it. Please clarify and make changes to the methods section as appropriate.
3. In the discussion section, it is not explained how this study shows a synergistic interaction between changes that occur on days 3, 14 and 21 and between a number of macrophages and fibroblasts in figures 1, 2 and 3 with no visible histopathologic changes. It's better to call it the calculation score.

**Minor**

1. In the method section, how much A *loe vera* contains? Explain the total solids content of the gel material used in the study.
2. In the results section, a description of the histopathological picture is explained in relation to the analysis results from figures 1,2 and 3. It would be easier to explain in the discussion section.
3. In the discussion section, please explain clearly the bioactive compounds contained in A *loe vera* other than glucomannan and acemannan. Are there other active ingredients? What do the authors mean treatment days 14 and 21? Describe the histopathological picture with figures 1,2 and 3.
4. In the introduction and abstract, *Aloe vera* intervention changes macrophages, fibroblasts and increases bioactive compounds and their potential for healing functional burns. The conclusion states that it is a potential pharmaceutical product. Please be consistent.

Is the work clearly and accurately presented and does it cite the current literature?

Yes

If applicable, is the statistical analysis and its interpretation appropriate?

Partly

Are all the source data underlying the results available to ensure full reproducibility?

Yes

Is the study design appropriate and is the work technically sound?

Yes

Are the conclusions drawn adequately supported by the results?

Yes

Are sufficient details of methods and analysis provided to allow replication by others?

Yes

Reviewer Expertise:

Physiology human, exercise, and much research-related use plants as herbal medicine

I confirm that I have read this submission and believe that I have an appropriate level of expertise to confirm that it is of an acceptable scientific standard, however I have significant reservations, as outlined above.

# Response

**Published:** 2022-03-09

**Authors:** Yunita Sari Pane

**Affiliations:** Universitas Sumatera Utara, Indonesia

Dear Prof Gusbakti Rusip,

 I really appreciate your willingness to review my manuscript. Here I explain more information to improve understanding in this article.

 Major:

1. We have assessed entirely during the present study, but I couldn't document all due to lack of memory byte card on the camera, So I took a few photos that can represented it and can be seen in figshare ( https://doi.org/10.6084/m9.figshare.17194973.v1)
2. We used the ANOVA test because in this study we used more than two groups with parametric data types
3. We have presented and correlated the changes that occur on days 3,14,21 in macrophages and fibroblasts that could be seen in Figures 1 and 2 and had been discussed in the discussion section.

Minor:

1. Aloe vera contained 10% of the total weight of the gel (see gel preparation)
2. In journal writing, repetition of words should be avoided, therefore we chose to explain in detail the number and comparison between groups in the results section. While in the discussion section we explained the comparison of our findings with other studies
3. Aloe vera has active ingredients other than acemannan and glucomannan, but based on the literature that we read, it proved that they could play a role in burn repair.
4. We did not state that aloe vera can be used as a pharmaceutical product in the conclusion section (see part conclusion). We only stated that aloe vera could accelerate the healing process as proven by this study.
